# PEO-sheathed liquid jets increase sample delivery stability for serial femtosecond X-ray crystallography

**DOI:** 10.1038/s41598-026-44308-8

**Published:** 2026-03-25

**Authors:** Mohammad Vakili, Saša Bajt, Johan Bielecki, Sabine Botha, Claire Cerniglia, Henry N. Chapman, Carsten Deiter, Raphael de Wijn, Katerina Dörner, Juncheng E, Petra Fromme, Raimund Fromme, Alfonso M. Gañán Calvo, Huijong Han, Phan Bao Ngoc Huynh, Chan Kim, Marco Kloos, Faisal H. M. Koua, Romain Letrun, José Domingo Meza-Aguilar, José M. Montanero, Katherine Morin, Tomas Popelar, Alejandro Rubio González, Christina Schmidt, Sunidhi Sharma, Egor Sobolev, Jui-Tung Tseng, Oleksii Turkot, Agnieszka Wrona, Jay-How Yang, Gayathri Yogaganeshan, Richard Bean

**Affiliations:** 1 European XFEL, Holzkoppel 4, 22869 Schenefeld, Germany; 2 Depto. de Ingeniería Mecánica, Energética y de los Materiales and Instituto de Computación Científica Avanzada (ICCAEx), Universidad de Extremadura, E-06006 Badajoz, Spain; 3 Departamento de Ingeniería Aeroespacial y Mecánica de Fluidos, Universidad de Sevilla, E-41092 Sevilla, Spain; 4 ENGREEN, Laboratory of Engineering for Energy and Environmental Sustainability, Universidad de Sevilla, 41092 Sevilla, Spain; 5 Biodesign Center for Applied Structural Discovery, Arizona State University, AZ 85287-5001 Tempe, USA; 6 School of Molecular Sciences, Arizona State University, AZ 85287-1604 Tempe, USA; 7 Department of Physics, Arizona State University, AZ 85287-1504 Tempe, USA; 8 Graduate Institute of Biochemistry, National Defense Medical University, Taipei, Taiwan; 9 Center for Free-Electron Laser Science CFEL, Deutsches Elektronen-Synchrotron DESY, Notkestr. 85, 22607 Hamburg, Germany; 10 The Hamburg Centre for Ultrafast Imaging, Luruper Chaussee 149, 22761 Hamburg, Germany; 11 Department of Physics, University of Hamburg, Luruper Chaussee 149, 22761 Hamburg, Germany

**Keywords:** Materials science, Optics and photonics, Physics

## Abstract

Viscoelastic jets can be generated by the polyethylene oxide (PEO) sheathing of an aqueous solution using double-flow focusing nozzles (DFFNs) and represent an efficient method to deliver samples that are dispersed in low and medium-viscosity liquids for X-ray diffractive imaging experiments. Due to their micrometre diameter and millimetre length, such jets can be used for pump–probe serial femtosecond crystallography (SFX) in order to access a timescale of a few tens of microseconds. This range is in between the previously achievable ranges accessible at XFELs (picoseconds-to-microsecond time delays) and synchrotrons (a few hundred µs to millisecond delays), respectively. Here, we demonstrate their effectiveness to deliver protein microcrystals (lysozyme and photosystem II) in buffer compositions of various viscosities for SFX and explore capabilities of triple-flow focusing nozzles (TFFNs) that incorporate PEO-sheathing to control challenging-to-jet viscous buffers for time-resolved diffusive mixing experiments.

## Introduction

Freely flowing liquid jets are an invaluable sample delivery method to X-ray diffractive imaging instruments where a constant sample replenishment is required^1^. Such highly controllable liquid jets provide minimal scattering background, a high signal-to-noise ratio, and can be operated at velocities of several tens of m/s, making them especially suitable for X-ray pulses with MHz repetition rates experienced at the European XFEL^2^.

Today, such liquid jets are usually generated by gas dynamic virtual nozzles (GDVN)^3^. Their development is based on a flow-focusing technique first published in 1998^4^. GDVNs employ a pressure-driven liquid (running through the first orifice) that is accelerated and converged by a coaxial gas-focusing (second orifice). With the introduction of a third orifice for a focusing sheath liquid^4,5^, double-flow focusing nozzles (DFFN) addressed the need to improve the operational stability of the nozzles^6^. Here, the sample stream is first focused by a coaxial, faster-flowing outer liquid (usually ethanol). The compound jet is then focused by coaxial gas flow as in the GDVN. Since the jet formation is assisted by the sheath liquid flow (which can run at a higher flow rate), the applied (inner) sample flow rate could be reduced (Table 1). The main benefit of a DFFN lies in the fact that it allowed the jetting of buffers that contain either a high amount of salts or polymeric stabilizing components, both influencing the fluid properties such as viscosity immensely and impeding the jetting capability.

**Table 1:** SFX statistics that compare the PSI crystal delivery between a GDVN and a DFFN (using EtOH as sheath liquid). The data (proposal no. 3111) was collected with the same nozzle at a 168.2 mm sample-AGIPD distance with nano-focused X-rays (9.3 keV, pulse energy *ca.* 1.7 mJ). Corresponding side view microscopy images of the jets can be found on Fig. 3.

Parameter	Sample	
	PSI	PSI+EtOH
Liquid flow rates^1^ (µL/min)	30+0	10+20
Helium flow rate (mg/min)	33.0	33.0
Jet velocity (m/s)^2^	45.2	44.2
Jet diameter (µm)	3.8	3.8
Intra-train rep. rate of pulses (MHz)	1.13	1.13
No. of pulses/train	352	352
No. of pulses/s	3,520	3,520
Total no. frames	1,095,822	1,100,034
No. of hits	11,997	3,365
Hit rate (%)	1.09	0.31
No. of indexed crystals	5,676	1,437
Prop. of indexable hits (%)	47.31	42.70
Total indexing rate (%)	0.52	0.13
Data collection time (min)^3^	9.14	36.25

^1^First value: sample flow, second value: sheath flow.

^2^Jet explosion method.

^3^The time it takes to acquire 10,000 indexed diffraction patterns under these conditions (considering the number of pulses, experienced hit rate and proportion of indexable hits).

We recently reported on the use of dilute polyethylene oxide (PEO) solutions as an alternative to ethanol for the sheath liquid in DFFNs^7^. The combination of PEO-sheathing of aqueous solutions with gas-focused liquid jets results in extremely high velocity gradients in the tapering meniscus that cause a coil-to-stretch transition of the polymer chains in the jet shell, resulting in viscous properties and an increased jet stability^8,9^. Such viscoelastic jets are much thinner and longer than their purely aqueous counterparts and their incorporation allows the nozzle to be operated at much lower applied flow rates than conventionally employed by GDVNs or DFFNs, where the latter uses an ethanol-sheath to stabilize and elongate the jet. Furthermore, extended jet lengths (>1 mm) have the potential to allow for pump–probe delays of > 10 µs in time-resolved SFX^10^, whereas conventional jets, that are around 200 µm long, limit the optical laser (pump) to X-ray (probe) spacing for delay times of <6 µs^7^. Moreover, the use of long jets enables the nozzle to be moved to a larger distance from the X-ray interaction region. This would prevent a back-splashing of debris from the beam-induced jet explosion onto the nozzle that may lead to nozzle clogging and experiment downtime.

**Fig. 1:**
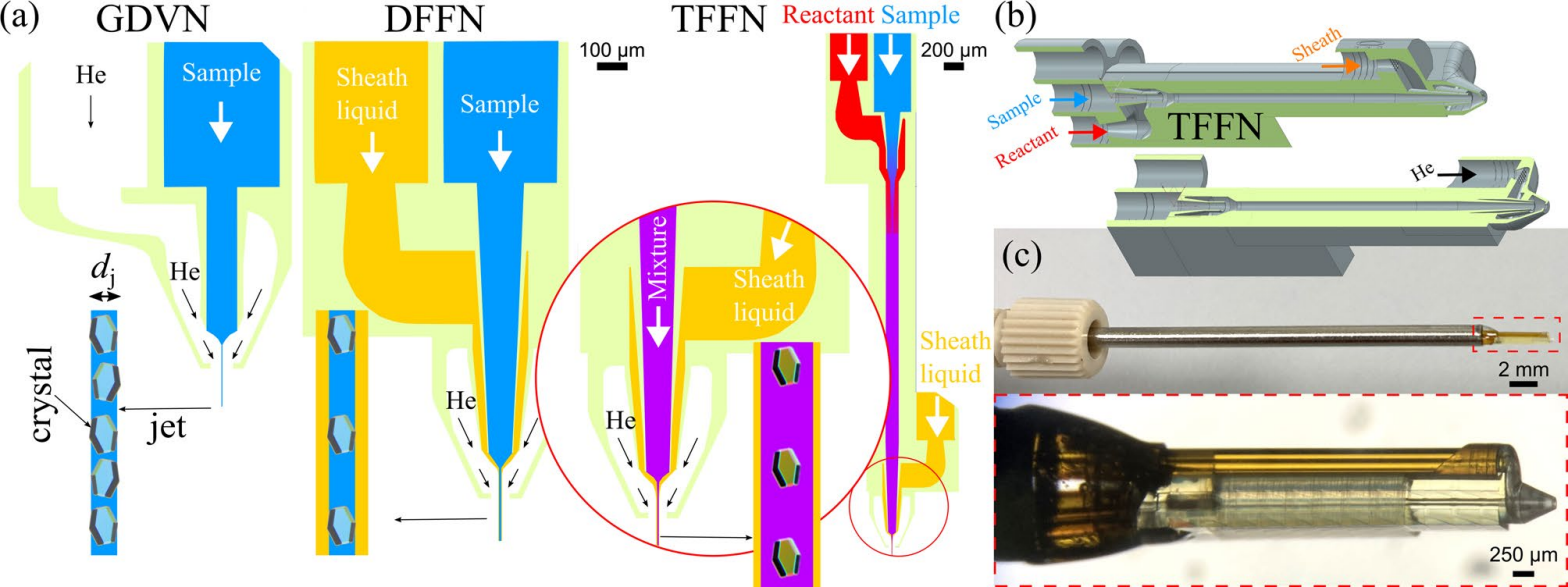
Overview of three nozzle types for liquid sample injection with indicated jets: gas dynamic virtual nozzle (GDVN), double-flow focusing nozzle (DFFN) and triple-flow focusing nozzle (TFFN). GDVNs accelerate the liquid sample via coaxial gas-focusing, which forms the thin liquid jet. DFFNs additionally employ a stabilizing sheath flow prior to gas-focusing. TFFNs are DFFNs combined with a micromixer, which sits upstream of the nozzle and allows the diffusion of reactants into the sample stream for time-resolved studies. (**b**) CAD representations of the type ’SLS’ TFFN. (**c**) Photos of an assembled TFFN.

Here, we describe SFX experiments with protein microcrystals sheathed with PEO solutions and discuss the hit rate and indexing rate statistics for a wide range of applied sample flow rates and buffer viscosities. Further, we describe a novel design that allows diffusive mixing of a reactant solution into the viscous buffer by adding a second sample channel to the DFFN, resulting in a single-piece, triple-flow focusing nozzle (TFFN) (Fig. 1). This design represents the next step in the evolution of previously presented mixing injectors^2,11^for mix-and-inject serial crystallography (MISC)^12–14^.

## Investigated samples

In addition to the well-characterized hen egg white lysozyme (HEWL), a ’model protein’^15^, we used PEO-jets to deliver photosystem II (PSII) microcrystals. PSII is a large multi-subunit membrane protein complex (*ca.* 700 kDa) that utilizes the solar energy to catalyze the light-induced charge separation that drives the water oxidation, thereby producing molecular oxygen, protons, and electrons. A charge separation across the membrane, *i.e.* membrane potential, is generated that drives reduction of NADP^+^ to NADPH and synthesis of ATP. These high energy products are then used for the subsequent CO_2_fixation and synthesis of carbohydrates^16–18^. Therefore, it remains as one of the most studied biological systems in X-ray crystallography with the main goal of unraveling its molecular mechanism^19,20^. However, because of its large unit cell, studying PSII by X-ray diffraction is highly challenging. One way to overcome poor diffraction was to use a post-crystallization dehydration procedure, which required the introduction of a high concentration cryo-protectant, e.g. polyethylene glycol (PEG), to shrink the unit cells that in turn improve the crystal packing and hence the diffraction quality^19^. However, this posed serious challenges in liquid jetting owing to the high viscosity introduced by the PEG (Table 2), that could be rectified by PEO-sheathing.

**Table 2:** Dynamic viscosity of various liquids and buffer solutions measured at 20 $\vphantom{0}^\circ$C. The LCP values are taken from^76^.

Solution	Viscosity (mPa s)
5 mM MES (pH 6.4), 50 mM MgSO_4_, 0.02% (w/v) *n*-dodecyl-*ß*-D-maltoside	0.63
5 mM MES (pH 6.4), 0.02% (w/v) *n*-dodecyl-*ß*-D-maltoside (PSI buffer)	0.73
0.2% (w/v) PEO100k	0.93
Water	0.97
10% (w/v) NaCl, 50 mM NaOAc (pH 3.5) (HEWL buffer)	1.10
7% (w/v) PEG1450	1.52
0.5 mM MES (pH 6), 100 mM KCl, 0.1% decyl-maltopyranoside, 10% glycerol	1.61
1% (w/v) PEO 100k	2.19
75 mM HEPES (pH 7.0), 20 mM MgCl_2_, 25% (w/v) PEG3350	3.96
15% (w/v) PEG4000	4.16
2% (w/v) PEO 100k	4.18
0.1 mM NaAc (pH 4.5), 3% glycerol, 20% PEG3350	5.09
0.1 M HEPES (pH 7.0), 0.88 M NaCl, 25% PEG1500	5.70
0.2% (w/v) PEO 100k, 20% (w/v) PEG6000	9.05
0.1 M MES pH (6.5), 0.2 M MgCl_2_, 25% PEG4000	10.82
20% (w/v) PEG6000	11.16
1% (w/v) PEO 100k, 20% (w/v) PEG6000	11.46
10% (w/v) NaCl, 50 mM NaOAc (pH 3.5), 20% (w/v) PEG6000 (HEWL2 buffer)	12.52
5% (w/v) PEO 100k	13.48
10% (w/v) PEO 100k	15.34
50 mM PIPES (pH 7.0), 5 mM CaCl_2_, 7.5% (w/v) PEG1450, 25% (w/v) PEG6000 (PSII buffer)	17.62
100 mM PIPES (pH 7.0), 10 mM CaCl_2_, 9% (w/v) PEG1450, 33% (w/v) PEG5000	41.45
Lipidic cubic phase (7:3 monoolein:water) at 0.3 1/s	5.7$\times$10^6^
Lipidic cubic phase (7:3 monoolein:water) at 75.4 1/s	3.6$\times$10^4^
15% (w/v) PEO 100k	249.65
20% (w/v) PEO 100k	474.33

For comparison, we used the 1.3 MDa photosystem I complex, which catalyzes the light-induced charge separation across the photosynthetic membrane and provides the electrons for the reduction of NADP^+^ to NADPH. PSI can be crystallized and delivered in extremely low-viscous media.

## Results and discussion

### SFX considerations and observations

SFX relies on the fact that the duration of the X-ray pulses generated by the X-ray free-electron laser (XFEL) is so short (*ca.* 50 fs), that diffracted photons exit the hit sample before the onset of significant radiation damage^21–23^. Diffraction is thereby detected from essentially undamaged molecules at or close to room temperature, *i.e.* near-physiological condition. In SFX terminology, a ‘hit’ denotes a diffraction pattern with the minimum number of detectable Bragg peaks^24^. One of a user’s biggest efforts during an SFX experiment is to maximize the hit rate, *i.e. *the ratio of X-ray pulses hitting a sample of interest to the available X-ray pulses^25^. The hit-images with the Bragg peaks are indexed, integrated, and scaled to provide the final three-dimensional structural information^26^. In serial crystallography, and due to random orientation of crystals, a high number of indexed frames is required to ensure data completeness with high redundancy to reconstruct the sample’s electron density with sufficient quality. A high hit rate together with a high proportion of indexable hits would reduce the time it takes to reach this goal and would allow the collection of multiple datasets during an allocated beamtime, for instance, various time points for a time-resolved study^27^. An example: many SFX experiments at SPB/SFX utilize an intra-train pulse repetition rate of 0.564 MHz, which provides 202 pulses per train (*i.e*. 2020 pulses/second). Assuming a (conservative) 1% hit rate and a 50% indexing rate, the theoretical time it takes to collect 10,000 patterns amounts to:

1 $$\begin{aligned} t_\text {theo} = \frac{(10\ 000 \times \frac{100}{1} \times \frac{100}{50})}{2020 \text { pulses/s}}=17 \text { min}. \end{aligned}$$

Similarly, operation at 1.13 MHz (with 352 pulses/train) would require a collection time of 9 min for the same amount of indexed frames.

At the SPB/SFX instrument, a crystal hit rate between 0.5–5.5% is often experienced when using liquid jets, owing to the unique X-ray pulse patterns and sample delivery constraints^2,28^. Usually, the sample reservoirs are prepared with a crystal concentration of 15% (v/v) (estimated by the pellet size in the sample vial/falcon after sedimentation), which already constraints the achievable hit rate. Loading slurries with higher crystal concentrations often leads to clogging in the feeding capillaries, nozzle constrictions and/or jetting instabilities. Additional losses that reduce the hit rate may occur due to some crystals getting stuck in the inline-filter, tubing connections, switching valve and/or reservoir. Moreover, smaller crystals, as well as only partially exposed crystals might result in very low diffraction, which is not captured properly on the detector.

The hit rate and indexing rate are important statistics that can be used to evaluate the sample quality, data collection strategy, and beamtime efficiency during SFX data collection and processing^26^. We illustrate these statistics for unsheathed, water-sheathed and PEO-sheathed low-viscous HEWL samples (Table 3), as well as for PEO-sheathed medium-viscous HEWL and PSII samples (Table 4). A linear relationship between the applied sample flow rate and crystal hit rate was observed: compared to GDVN operation, the PEO-DFFN allowed for the reduction of the HEWL flow rate by 33% which in turn led to a decrease of the hit rate by 33%. Moreover, we observed an increase in hit rate of 73% by increasing the beam size from the nano-sized beam to a de-focused, micron-sized beam. Using this relation, we correlate that stable hit rates of 3–5% can be achieved routinely with the micro beam, which allows full datasets to be collected in under 5 minutes (see eq. 1). It is worth noting that the use of nanocrystals in combination with similar jets can result in hit rates $\ge$80% due to multi-crystal exposure^29^.

**Table 3:** SFX data collection and refinement statistics, Part I.

Parameter	Sample				
	HEWL1^3^	HEWL1	HEWL1+H_2_O	HEWL1+PEO	HEWL1+PEO
Liquid flow rates^1^ (µL/min)	30+0	30+0	20+10	20+10	10+20
Helium flow rate (mg/min)	27.0	27.0	30.9	28.5	28.4
No. of pulses/train	202	202	100	352	352
No. of pulses/s	2,020	2,020	1,000	3,520	3,520
Total no. frames	1,205,598	1,808,196	594,900	1,052,298	1,035,450
No. of hits	41,769	36,231	6,473	14,207	4,704
Hit rate (%)	3.46	2.00	1.09	1.35	0.45
No. of crystals	48,578	40,277	4,151	13,225	4,568
No. of indexed crystals	38,666	34,126	3,975	12,605	4,362
Prop. of indexable hits (%)	92.57	94.19	61.41	88.72	92.73
Total indexing rate (%)	3.21	1.89	0.67	1.20	0.42
Data collection time (min)^2^	3.57	4.37	24.94	3.95	11.24
Unit cell lengths *a*,*b*,*c* (Å)	79.19/79.21/38.08	79.20/79.20/38.08	79.14/79.04/37.94	79.02/79.03/37.91	79.09/79.06/37.90
Unit cell angles$\alpha$,$\beta$,$\gamma$(°)	90.00/90.00/90.00	90.00/90.00/89.99	90.09/89.90/89.92	90.01/89.98/89.99	90.05/89.95/89.96
Resolution (Å)	19.23-1.70	19.23-1.70	19.01-1.70	19.01-1.70	19.01-1.70
	(1.76-1.70)	(1.76-1.70)	(1.76-1.70)	(1.76-1.70)	(1.76-1.70)
Signal-to-noise ratio	8.42 (2.49)	8.44 (3.81)	3.46 (2.76)	4.80 (3.52)	3.20 (2.52)
Completeness (%)	100.00 (100.00)	100.00 (100.00)	100.00 (100.00)	100.00 (100.00)	100.00 (100.00)
Redundancy	280.99 (189.50)	219.74 (148.60)	44.10 (30.40)	111.27 (76.00)	40.53 (27.90)
*R*_split_ (%)	10.84 (31.10)	12.39 (23.61)	37.47 (47.30)	27.31 (39.47)	42.68 (54.94)
CC_1/2_ (%)	97.97 (83.77)	97.59 (88.68)	74.95 (64.25)	86.87 (73.69)	70.14 (53.52)
CC* (%)	99.49 (95.48)	99.39 (96.95)	92.57 (88.45)	86.42 (92.12)	90.80 (83.50)

^1^First value: sample flow, second value: sheath flow.

^2^The time it takes to acquire 10,000 indexed diffraction patterns under these conditions (considering the number of pulses, experienced hit rate and proportion of indexable hits).

^3^Using a larger (3.5 µm wide) X-ray beam (de-focused nano-KB mirrors). Every other sample (also the ones shown in Table 4) was probed with a 600 nm wide beam (focused nano-KB mirrors).

**Table 4:** SFX data collection and refinement statistics, Part II.

Parameter	Sample			
	HEWL2+PEO	HEWL2+PEO	HEWL2+PEO	PSII+PEO^3^
Liquid flow rates^1^ (µL/min)	5+12.5	10+12.5	20+12.5	20+10
Helium flow rate (mg/min)	20.9	23.5	29.8	19.9
No. of pulses/train	352	352	352	202
No. of pulses/s	3,520	3,520	3,520	2,020
Total no. frames	3,140,046	3,140,748	3,141,450	1,321,776
No. of hits	5,067	12,617	18,128	6,166
Hit rate (%)	0.16	0.40	0.58	0.47
No. of crystals	5,407	12,371	16,961	697
No. of indexed crystals	3,745	8,501	11,664	689
Prop. of indexable hits (%)	73.91	67.38	64.34	11.17
Total indexing rate (%)	0.12	0.27	0.37	0.05
Data collection time (min)^2^	39.40	17.49	12.75	158.3
Unit cell lengths *a*,*b*,*c* (Å)	78.57/78.58/37.99	78.57/78.57/37.98	78.59/78.58/37.97	129.45/227.22/305.27
Unit cell angles$\alpha$,$\beta$,$\gamma$(°)	90.10/89.89/89.92	90.05/89.93/89.98	90.03/86.96/90.00	90.08/90.05/90.06
Resolution (Å)	19.01-1.70	19.01-1.70	19.01-1.70	15.24-4.50
	(1.76-1.70)	(1.76-1.70)	(1.76-1.70)	(4.66-4.50)
Signal-to-noise ratio	3.97 (1.87)	5.60 (2.63)	5.96 (3.00)	1.85 (1.51)
Completeness (%)	100.00 (100.00)	100.00 (100.00)	100.00 (100.00)	83.58 (86.00)
Redundancy	63.22 (43.10)	133.64 (90.70)	159.96 (108.00)	6.84 (6.00)
*R*_split_ (%)	25.62 (46.28)	18.21 (31.69)	17.60 (29.64)	56.99 (79.30)
CC_1/2_ (%)	89.31 (75.52)	94.11 (84.49)	94.25 (86.39)	60.47 (25.42)
CC* (%)	97.13 (91.69)	98.51 (95.71)	98.51 (96.28)	86.81 (63.67)

^1^First value: sample flow, second value: sheath flow.

^2^The time it takes to acquire 10,000 indexed diffraction patterns under these conditions (considering the number of pulses, experienced hit rate and proportion of indexable hits).

^3^Data from proposal no. 8756 from February 2025 (12.5 keV, nano-focused beam).

With the nano-focused beam, we further achieved a PSII hit rate of 0.5% with an 11% proportion of indexable hits. Albeit very low, these values imply a required data collection time of 2.6 hours to reach 10,000 indexed patterns. To this date, this represents the best data collection statistics for PSII microcrystals measured at the European XFEL with a liquid jet, thus paving the way for future use of the PEO-sheathed sample delivery method for time-resolved SFX with viscous sample buffers.

### SFX hit rate simulations

**Fig. 2:**
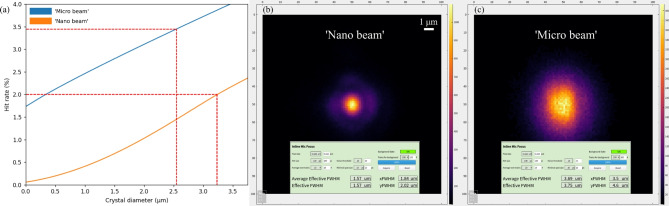
(**a**) Theoretical single-crystal hit rates for a liquid jet (here: *d*_j_=3.8 µm) delivery. The two X-ray beam sizes resulted from different nano-KB optics alignments, which provided beam sizes of 3.5 µm $\times$ 3.5 µm (de-focused nano-KB mirrors, blue) and *ca.* 600 nm $\times$ 600 nm (focused nano-KB mirrors, orange), respectively. The data is shown for a 0D Poisson point distribution. The red-dotted lines indicate the achieved 3.46% and 2.00% hit rates for ’Micro beam’ and ’Nano beam’ exposure on the HEWL1 sample (see Table 3), respectively, and are in good agreement with the actual probed crystal sizes (measured to be 2–4 µm via optical microscopy, see Fig. 12). (**b**) and (**c**) show images of a scintillating YAG:Ce crystal at the sample interaction region that were captured with the inline microscope during the respective X-ray focusing procedures.

To model the achievable hit rates under our experimental conditions, we simulated single-crystal hit rates as a function of crystal size and X-ray focus size. For this, we used an approximate hard sphere probability distribution^30^ to account for excluded volume effects in the number density of finite-sized crystals in the beam. In addition, the 1D hard rod probability distribution was extracted from the literature^31^. Fig. 2a shows a plot of the Poisson results, which are in good agreement with the actually probed HEWL crystals. The parameters for the simulation are based on typical experimental conditions: a 3.8 µm wide liquid jet, running at 45 m/s, and containing 6$\times$10^8^ crystals/mL. The true hit rate can be approximated by the Poisson model at small crystal sizes $d_{\text {crystal}}\ll d_{\text {jet}}$ and by the hard rod model at larger crystal sizes $d_{\text {crystal}} \ge d_{\text {jet}}$. Fig. 2b and c show images of a scintillating YAG:Ce crystal at the sample interaction region during X-ray alignment/focusing as captured with the inline microscope. These results are a convolution of the point spread function (PSF) of the optical system and intensity distribution from the YAG:Ce screen. The real focus size is approximated by convolving the ideal PSF, measured from monochromatic Rayleigh scattering from a <50 nm object, with a range of focal sizes while taking the spectral shape of the YAG emission into account.

### Compound jets with biological samples

PEG (polyethylene glycol) is a hydrophilic, non-ionic and bio-compatible polyether comprised of the repeating unit (-CH_2_-CH_2_-O-)_*n*_. Typically, polymers with molecular weights below 20,000 g/mol are referred to as PEG, whereas those with molecular weights above 20,000 g/mol are referred to as PEO (polyethylene oxide)^32^. PEGs have an enormous importance in the food and pharmaceutical industry and due to their strong volume exclusion and dehydrating effects^17,19^are often utilized during the preparation of biological samples for diffractive X-ray imaging methods such as serial crystallography^33^.

To provide an ideal environment, many protein crystals, especially those with large unit cells (such as photosystem II and cytochrome c oxidase) use buffers with PEG concentrations of $\ge$20% (w/v), which significantly increases the viscosity (see Table 2). Viscous liquids, however, are difficult to jet with a GDVN as they resist the shear and acceleration from the focusing gas, which makes a DFFN the preferred choice to provide compound jets. The concept of double-flow focusing to control nanometre-scale volumes of liquids has been around for more than two decades^4,5^, has been adapted to provide nozzles for SFX in 2017^6^, and designs are constantly being refined^11,34^.

#### Ethanol-sheathed liquids

Currently, ethanol-sheathed liquid jets at SPB/SFX are generated with a 3D printed DFFN, which provides a liquid orifice of $D_{\text {i}}$=75 µm, gas orifice of $D_{\text {gas}}$=70 µm, and an orifice-to-orifice spacing of $H_{\text {ig}}$=70 µm (’JKMH-8.11’)^11^. Latest design iterations also offer 30 µm (type ’Q’) and 100 µm (type ’N’) liquid orifices to cater to a wide range of sample types^35^. In general, DFFNs may also be operated as GDVNs simply by not applying a sheath liquid flow.

**Fig. 3:**
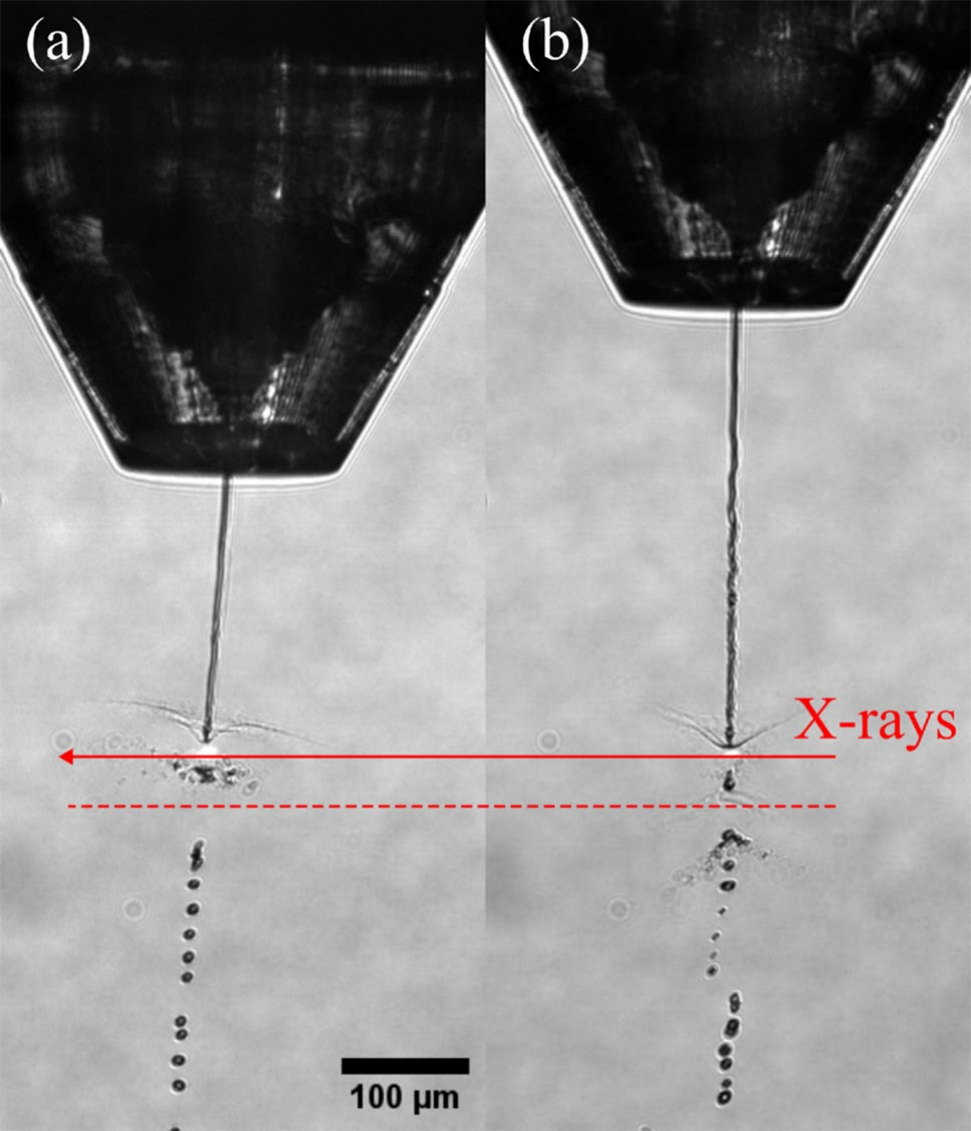
Microscopy images (10$\times$ magnification, NA 0.28, pixel size $\sim$1.26 µm) showing PSI crystal delivery (proposal no. 3111) in low-viscous buffers on the SPB/SFX instrument using a type JKMH-8.11 DFFN. The second X-ray pulse of the train is indicated with a red arrow. The gap generated by the first pulse (the red dotted line depicts its center) has traveled *ca.* 40 µm in the 887 ns between these two pulses (*i.e.* 1.13 MHz). The images show *ca.* 4 µm wide jets of PSI crystal-laden buffer (without PEG) generated with the same nozzle: (**a**) GDVN mode (*i.e.* no sheath flow running) and (**b**) with additional ethanol sheath flow. The applied flow rates were (**a**) $Q_i=30$ µL/min and (**b**) $Q_i=10$ µL/min plus $Q_o=20$ µL/min, respectively. The helium flow rate was $\dot{m}_g=33$ mg/min in both cases. As shown in Table 1, the increased jet length and stability comes at the expense of crystal hit rate due to a required reduction of the sample flow rate to accommodate to high repetition rate pulses.

While ethanol-sheathing leads to an increased jet length and stability (Fig. 3), it is usually accompanied by a reduction of the sample flow rate to allow the generation of thin/fast jets compatible for megahertz serial crystallography^36,37^. This flow rate reduction in turn reduces the hit rate, which implies that the data collection time has to be increased to achieve the same number of indexable hits. Therefore, the overall sample consumption remains the same. As seen in Table 1, a 3-fold reduction of the sample flow rate in DFFN-mode lead to a 3.5-fold decrease in hit rate. This linear relation was also documented in further experiments with the PEO solution as sheath flow liquid (Table 3).

#### PEO-sheathed liquids

**Fig. 4:**
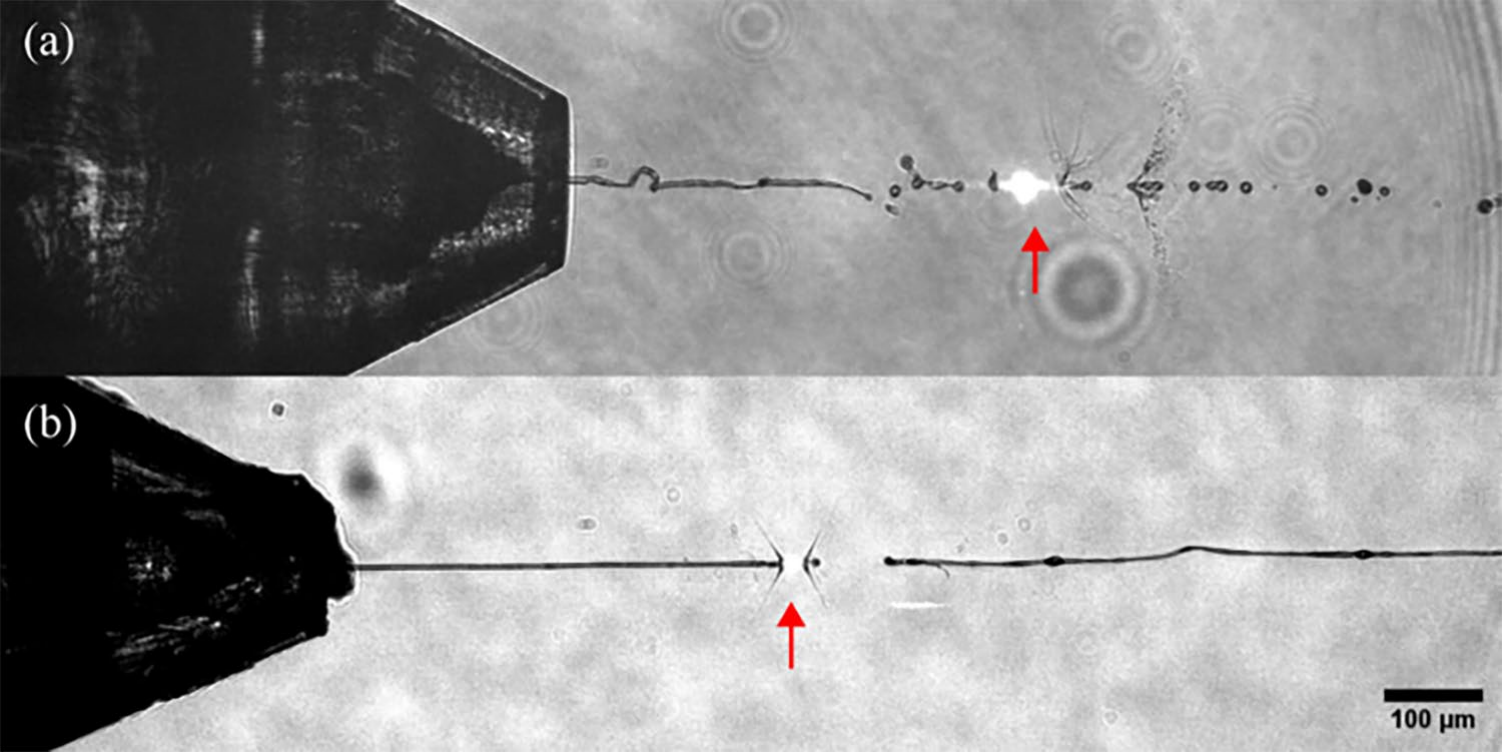
Microscopy images (10$\times$ magnification, NA 0.28, pixel size $\sim$1.26 µm) showing PSII crystal delivery in medium-viscous buffers on the SPB/SFX instrument using a DFFN. The interaction position with the X-rays is indicated with a red arrow. The images show crystal-laden buffer surrounded by ethanol (a, proposal no. 3333) and the viscoelastic shell (b, proposal no. 8756). The jets (*d*_jet_ $\sim$ 4 µm) were generated with $Q_i=25$ µL/min and $Q_o=40$ µL/min (**a**) and $Q_i=20$ µL/min and $Q_o=10$ µL/min (**b**), respectively. The mass flow rate was $\dot{m}_g=30$ mg/min (**a**), and 19 mg/min (**b**), respectively. While the EtOH-sheathed jet wiggles strongly, the PEO-sheath enables a long and steady jet. Table 4 compares the hit rate statistics.

**Fig. 5:**
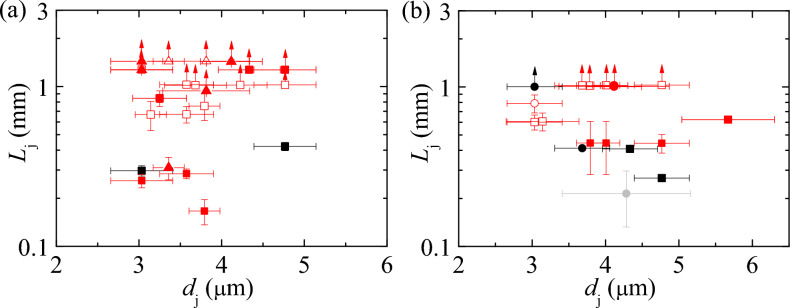
Jet length $L_\text {j}$ versus jet diameter $d_\text {j}$ for a compound jet generated with a type N9 double-flow focusing nozzle. Panels (**a**) and (**b**) show experimental realizations without and with beam-jet interaction, respectively. The core jet liquid was either water (squares), 10% (w/v) NaCl, 50 mM NaOAc (pH 3.5), 20% (w/v) PEG6000 (triangles) [panel (a)] or 10% (w/v) NaCl, 50 mM NaOAc (pH 3.5) (circles) [panel (b)]. The grey, black and red symbols correspond to jets without a sheath, with a water sheath and with a 1% (w/v) PEO100k solution sheath, respectively. The hollow red symbols correspond to experimental realizations in which the jet suffered from the whipping instability. The arrows indicate that the jet length was larger than the stated value because the jet length exceeded the size of the image.

Upon increasing the viscosity of the sample buffer (>5 mPa s), ethanol as sheath liquid struggles to stabilize the sample stream in a compound jet (Fig. 4). On the contrary, aqueous PEO100k solutions at 1% (w/v) enable the production of jets with lengths larger than 1 mm, exceeding the size of the image (Fig. 5), which is more than 4$\times$ longer than that of the regular DFFN jets under similar conditions (see Fig. 3b). The whipping instability that typically arises in such long jets can be eliminated by selecting the gas and liquid flow rates appropriately. Besides the low-viscous core liquids (viscosity <5 mPa s), especially medium-viscosity liquids (5–50 mPa s, see Table 2) showed great compatibility with PEO-sheathing. Parenthetically, we consider liquids with a viscosity of $\ge$5,000 mPa s (such as lipidic cubic phase media^38,39^ or grease carriers^40,41^) as high-viscosity liquids. Such media are not compatible with DFFNs and require a hydraulic stage to slowly extrude the sample stream^42,43^.

The PEO-water jets were generated with a type ’N9’ double-flow focusing nozzle, that was described in^7^. Its design provides a liquid orifice of $D_{\text {i}}$=75 µm, a gas orifice of $D_{\text {gas}}$=60 µm, and an increased orifice-to-orifice spacing of $H_{\text {ig}}$=130 µm. Details on the 3D printing procedure for device preparation and device assembly can be found elsewhere^2^. A detailed description of the PEO background signal can be found in the section ’PEO conformation and X-ray profile’, while the formation of jet stabilization is discussed in ’PEO stretching and relaxation’.

**Fig. 6:**
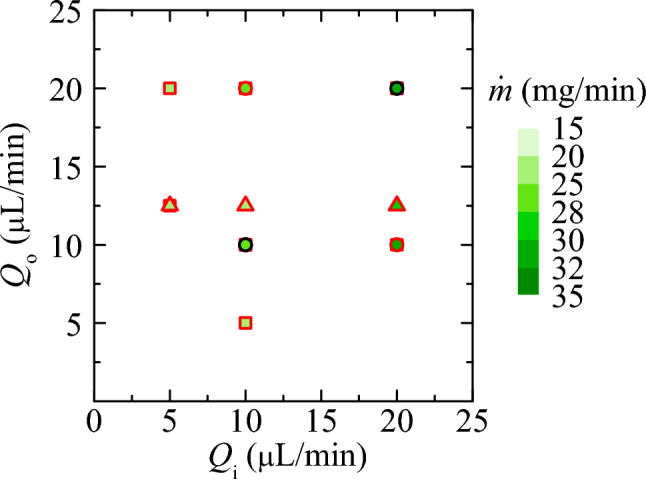
Applied core ($Q_i$) and sheath ($Q_o$) flow rates corresponding to the experimental realizations in Fig. 5. The jet core liquid was either water (squares), 10% (w/v) NaCl, 50 mM NaOAc (pH 3.5), 20% (w/v) PEG6000 (triangles) or 10% (w/v) NaCl, 50 mM NaOAc (pH 3.5) (circles). The symbols with black and red edges correspond to experiments with a sheath of water and aqueous PEO100k solutions at 1% (w/v), respectively. The color scale indicates the helium mass flow rate.

**Fig. 7:**
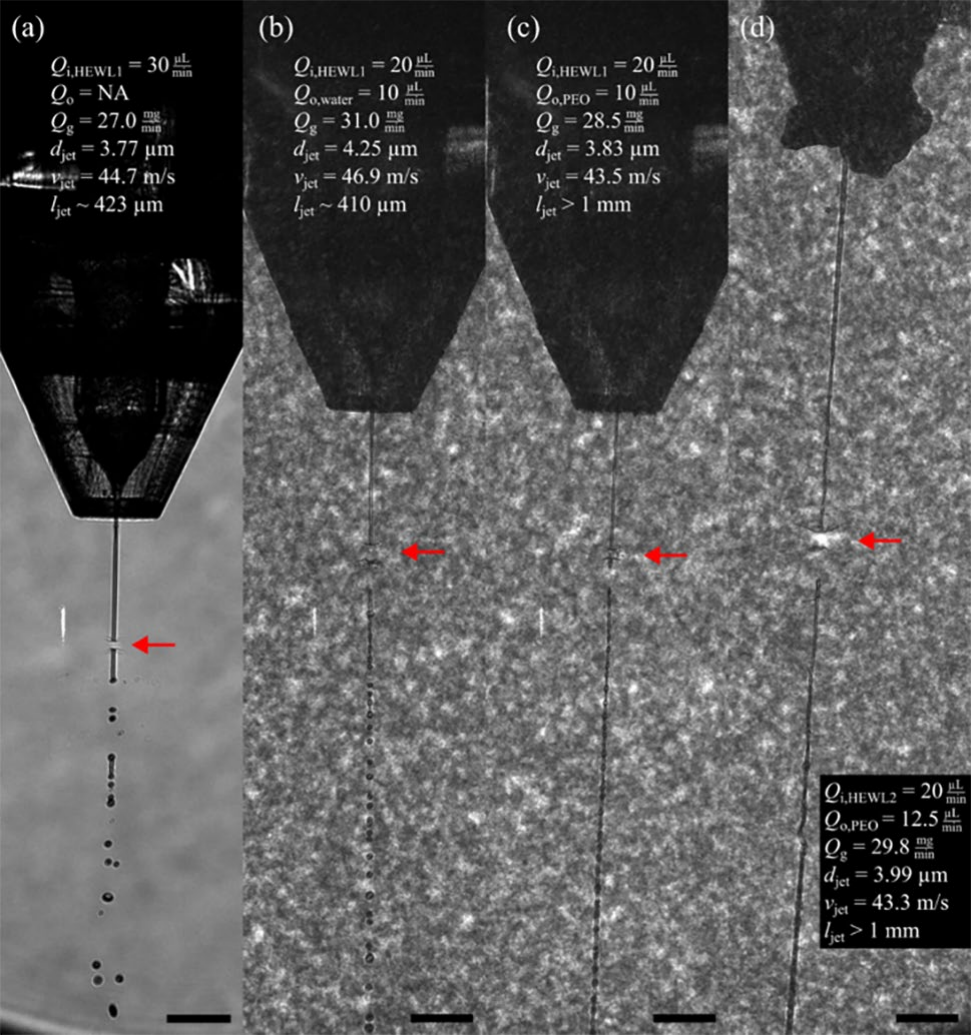
Microscopy images (10$\times$ magnification, NA 0.28, pixel size $\sim$0.65 µm) of HEWL crystal delivery on the SPB/SFX instrument (proposal no. 8796). The X-ray interaction position (red arrow), applied flow rates, jet characterization parameters (diameter, velocity and length) and 100 µm scale bars are denoted. Details on X-ray parameters can be found in Table 5.

Figure 6 shows the inner (core) flow rate, $Q_i$, and the outer (sheath) flow rate, $Q_0$, used in the experiments. Fig. 7 shows exemplary microscopy images of the X-ray interaction with the PEO-sheathed jets. Here, slight variations in observable gap width result from different effective pulse energies. The dynamics of a water jet explosion and the width of a jet gap as a function of pulse energy is investigated in^44^. A comprehensive depiction of all jetting parameters, X-ray parameters and jet characterization results can be found in Table 5.

**Table 5:** Experimental jetting parameters set at the SPB/SFX interaction region upstream (IRU) sample chamber (*p*$$\approx$$$$10^{-3}$$ mbar), X-ray parameters and jet characterization results. The ’effective pulse energy’ considers the measured pulse energy at the XTD2 X-ray gas monitor, the selected attenuators at the XTD9 tunnel (total transmission) and that the transmission with the nano-KB mirrors is about 60%. The results from the ’scaling law’ are obtained by applying the jet velocity prediction formula from^2^. Jets with velocities of *v*$$\ge$$25 m/s are fast enough for 0.564 MHz operation, while 1.13 MHz operation is compatible with jets with *v*$$\ge$$45 m/s.

Sample flow		Sheath flow		Total *Q*	Dilution	He flow	X-ray parameters							Jet explosion-based characterization			Scaling law		Microscopy
Sample	Flow rate [μL/min]	Solution	Flow rate [μL/min]	[μL/min]	x-Fold	Flow rate [mg/min]	Intra-train rep. rate [Mega 1/s]	No. pulses/train	Photon energy [keV]	Pulse energy [mJ]	Transm. [%]	Eff. pulse energy [μJ]	Detector distance [mm]	Displacement between two pulses [μm]	Jet velocity [m/s]	Jet diameter calc. [μm]	Jet velocity theo. [m/s]	Jet diameter theo. [μm]	Jet diameter [μm]
HEWL	30.0	NA	0.0	30.0	1.00	27.0	0.564	202	11.0	1.49	2	18	124.3	79.3	44.7	3.77	46.2	3.71	4.28±0.87
HEWL	20.0	Water	10.0	30.0	1.50	31.0	1.128	100	11.0	0.83	47	234	124.3	41.6	46.9	3.68	48.9	3.61	3.68±0.38
HEWL	20.0	PEO 1%	10.0	30.0	1.50	28.5	1.128	352	11.0	0.75	47	212	124.3	38.5	43.5	3.83	47.2	3.67	4.12±0.19
HEWL	10.0	PEO 1%	20.0	30.0	3.00	28.4	1.128	352	11.0	0.74	47	209	124.3	35.8	40.3	3.97	47.1	3.68	4.12±0.38
HEWL2	5.0	PEO 1%	12.5	17.5	3.50	20.9	1.128	352	11.0	0.89	22	117	124.3	39.5	44.6	2.89	46.4	2.83	3.47±0.38
HEWL2	10.0	PEO 1%	12.5	22.5	2.25	23.5	1.128	352	11.0	0.91	22	120	124.3	37.7	42.5	3.35	46.3	3.21	3.68±0.38
HEWL2	20.0	PEO 1%	12.5	32.5	1.63	29.8	1.128	352	11.0	0.88	22	116	124.3	38.4	43.3	3.99	47.3	3.82	4.33±0.75
PSII	20.0	PEO 1%	10.0	30.0	1.50	19.9	0.564	202	12.5	1.10	43	284	126.6	57.9	32.7	4.42	40.8	3.95	4.41±0.63
PSII	25.0	EtOH	40.0	65.0	2.60	30.0	0.564	202	9.3	1.75	100	1050	166.0	78.1	44.0	5.60	41.2	5.79	5.21±0.19
PSI	20.0	EtOH	10.0	30.0	1.50	33.0	1.128	352	9.3	2.60	40	624	168.2	39.2	44.2	3.79	44.2	3.80	3.02±0.45
PSI	30.0	NA	0.0	30.0	1.00	33.0	1.128	352	9.3	2.57	40	617	168.2	40.1	45.2	3.75	44.2	3.80	2.81±0.32

#### Mix-sheath-inject

**Fig. 8:**
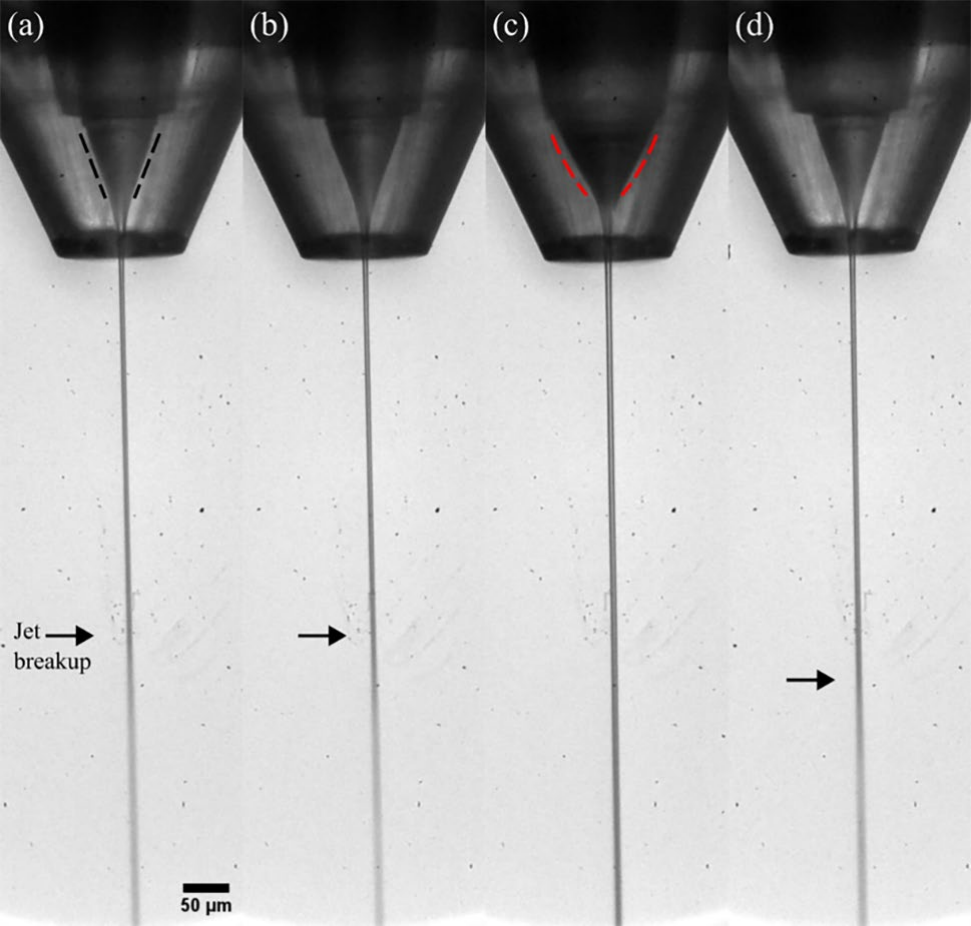
Laboratory microscopy images (20$\times$ magnification, NA 0.42, pixel size $\sim$0.97 µm) showing unsheathed and PEO-sheathed jets generated by a TFFN ($\dot{m}_g=30$ mg/min in each case) in a 0.5 mbar environment. (**a**) Water jet generated by applying $Q_i=30$ µL/min to the sample channel (the other channels were plugged). The V-shaped meniscus is indicated with black-dotted lines. (**b**) Two-channel water:water mixing with $Q_i=15$ µL/min and $Q_r=15$ µL/min. (**c**) A triple-flow focusing operation with a PEO-sheathed water:water jet with $Q_i=15$ µL/min, $Q_r=15$ µL/min, and $Q_o=15$ µL/min, resulting in an U-shaped meniscus (red-dotted lines). In (**d**), the same total flow rate $Q_i+Q_r=45$ µL/min was chosen as in (**c**), to demonstrate that the increased jet length does not arise from the higher total flow rate and that the absence of the viscoelastic fluid leads to a much shorter jet. The black arrows indicate the jet lengths, which here are not clearly visible as the LED illumination in combination with a 5 µs camera exposure time and a 10,000 1/s frame rate captured motion blurred droplet regimes.

To further enable time-resolved studies of structural changes triggered by diffusive mixing for challenging-to-jet buffers, we introduced a second liquid channel to the N9 DFFN design. By doing this, we realized a compact single-piece injector (type ’SLS’) that allows the micromixing of two liquids (*i.e.* the first, hydrodynamic focusing of the sample stream), which is followed by a PEO-sheath flow-focusing (second focusing) and a subsequent gas focusing (third focusing): a triple-flow focusing nozzle (TFFN) (Fig. 8). The length of the mixing channel is 3.5 mm, the inner diameter is 125 µm and we expect to probe delays between 10–30 ms with this geometry^2^, while longer delays can be achieved with a capillary bridging between separate micromixer and nozzle devices.

## Conclusions and outlook

PEO-sheathing of low- and medium-viscous solutions lead to superstable, low-background (the radial intensity profiles from the SFX experiments showed no trace of PEO in the jet) liquid jets for reliable microcrystal delivery across the beam of an X-ray free-electron laser. Especially in combination with high-PEG-laden crystal buffers, which exert high viscosities that are usually not suited for GDVN nor ethanol-DFFN operation, the PEO-jets demonstrated a significantly increased jet stability, allowing an improved sample-beam overlap.

Supported by theoretical formulations and simulations, our experimental data revealed that the use of PEO-sheathing of liquid jets in combination with a micron-sized beam focus in the range of the crystal size and jet size, provides an improved SFX data collection strategy for liquid samples. It is expected that stable crystal hit rates of 3–5% can be achieved routinely, which allows full datasets to be collected in under 5 minutes with megahertz intra-train pulse repetition rates.

Finally, we constructed an additional channel and orifice to our DFFN design, which resulted in a compact triple-flow focusing nozzle (TFFN) ready to use for the user community^35^. With such TFFNs, the high jet stability known from DFFNs can be introduced to micromixing injectors for time-resolved studies at XFELs, even when relying on challenging-to-jet buffer compositions or when studying an enzymatic reaction that utilizes two substrates, *i.e.* bi-bi reaction^45^.

## PEO structure and dynamics

### PEO conformation and X-ray profile

**Fig. 9:**
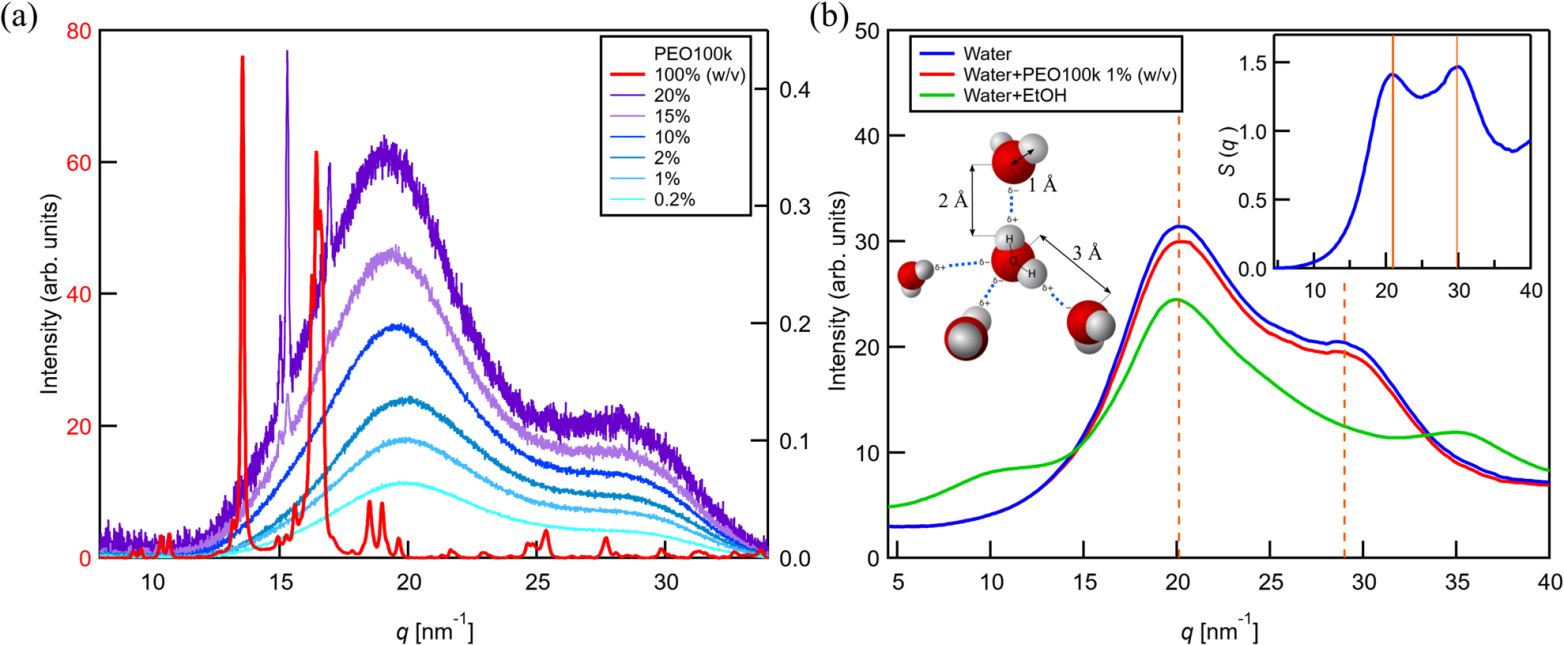
(**a**) XRD scattering curves of crystalline PEO (red) collected with an 8.04 keV laboratory source, showing high ordering of collapsed chains (red curve) that exert intramolecular hydrogen bonds. The two prominent intensity peaks at *q*=13.5 nm^−1^ and 16.5 nm^−1^ (4.6 Å and 3.8 Å) vanish upon dissolution in water as shown in the violet to blue curves (chains are relaxed, see Fig. 10b). (**b**) Scattering curves (*I*(*q*); averaged over 211200 frames that were collected on the SPB/SFX instrument) of various liquid (compound) jets. The data was generated with a photon energy of 11 keV (9.3 keV for the green curve). The applied liquid flow rates were $Q_{i, \text {water}}=20$ µL/min plus $Q_{o,\text {water}}=20$ µL/min (blue curve), $Q_{i,\text {water}}=20$ µL/min plus $Q_{o,\text {PEO}}=20$ µL/min (red curve, $c_{\text {PEO,eff}} \approx 0.5\% \text {(w/v)}$), and $Q_{i,\text {water}}=25$ µL/min plus $Q_{o,\text {EtOH}}=40$ µL/min (green curve), respectively. The helium mass flow rate was $\dot{m}_g=$32 mg/min in all cases. The inlay shows the *S*(*q*) plot for the water jet curve.

PEO forms strong hydrogen bonds with water, making it highly hydrophilic. In the absence of water, intramolecular hydrogen bonds are formed among PEO chains. Powder diffraction (using $\hbox {Cu } \hbox {K}{\alpha }$ radiation) on PEO at 100% solids (Fig. 9a) revealed two prominent intensity maxima at 2$\theta$ = 19.12$\vphantom{0}^\circ$ (*q*=13.5 nm^−1^; real space distance: 4.6 Å) and 23.25$\vphantom{0}^\circ$ (*q*=16.5 nm^−1^; 3.8 Å) that are assigned to the (120) and (032) planes of crystalline PEO^46–50^, respectively. PEO crystallizes in a monoclinic (primitive) lattice with space group *P*2_1_/*a* and shows helical symmetry D7 (the polymer chain makes 7 repeat units in two full turns)^51,52^. XRD measurements of aqueous solutions revealed that PEO concentrations as high as 10% (w/v) already do not have any signals beyond the hydrogen bond signals (see below). This indicates that, when in solution, the chains are highly relaxed, with limited interaction between the oxygen atoms of the same chain.

For the low-concentration sample, peak positions of 19.9 and 28.5 nm^−1^, respectively, were found. Upon increasing the PEO concentration to 20% (w/v), a slight shift of the first peak to 19.1 nm^−1^ can be observed, which could hint to longer intermolecular water distances and/or the emergence of intramolecular PEO hydrogen bonds (the chains collapse). Additionally, we see the emergence of crystallinity at $c\ge$15% (w/v) with strong signals at 14.9 and 15.2 nm^−1^ (2$\theta$=21.11 and 21.57$\vphantom{0}^\circ$). Aqueous solutions with PEO100k concentrations higher than 20% (w/v) could not be prepared.

The radial intensity profiles from the SFX experiments (Fig. 9b), indicate no trace of PEO in the jet, maintaining the low-background property known from pure water jets. The probed water jet and water/PEO jet (*d*_jet_ $\sim$ 4 µm) solely show prominent signals at 20.1 and 29.0 nm^−1^ (orange dotted line) that arise from hydrogen bonds between water molecules. While the first signal corresponds to the oxygen-oxygen distance between neighboring molecules (*ca.* 3 Å), the latter arises from the hydrogen bond length between an oxygen and a hydrogen atom (*ca.* 2 Å). The covalent O-H bond within a single molecule (bond length *ca.* 1.09 Å^51^) is beyond the resolution limit. The water/ethanol jet (*d*_jet_ $\sim$ 5 µm) has a broad maximum at 10 nm^−1^ (6.3 Å) due to water clustering in water-alcohol mixtures^53,54^ and at 35 nm^−1^ (1.8 Å) due to intermolecular hydrogen bonds.

The temperature-dependent structure factor, *S*(*q*), was obtained as detailed in^55^. The peak positions of the scattering curves in Fig. 9b are consistent with the 288–298 K peak positions (orange ribbons) from the water structure study from Sellberg *et al.*, hence, indicating a $\sim$1% jet diameter reduction due to evaporative cooling^56,57^.

**Fig. 10:**
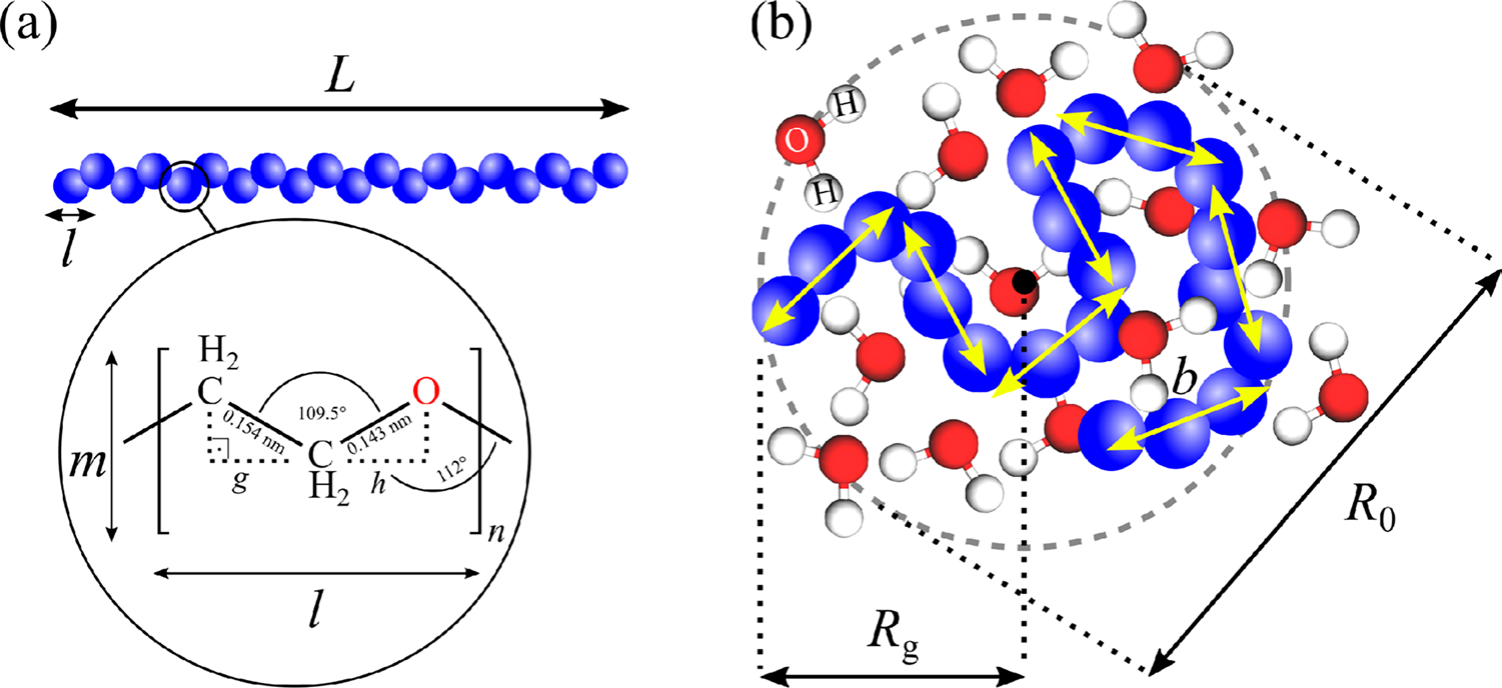
PEO structure models. (**a**) A general polyethylene oxide backbone. While the persistent chain (stretched) shows large Kuhn lengths *b*, the flexible chain **(b**) (e.g. in solution) shows a higher degree of entanglement with short Kuhn lengths. The ratio of $R_0$ to $R_\text {g}$ is ${6^{1/2}}=2.45$ for an ideal chain and therefore close to the diameter to radius ratio from spheres.

For a better visualization of the PEO conformation in the jet shell, we first consider a fully-stretched polymer in all-trans conformation (Fig. 10). The length *L* will be the product of the number of monomers (degree of polymerization) *n* and the monomer length *l*:

2 $$\begin{aligned} L = n\, l. \end{aligned}$$

With a C-C bond length of 0.154 nm, a C-O bond length of 0.143 nm^51^, and assuming a C-C-O angle of 109.5$\vphantom{0}^\circ$ (ideal tetrahedral) and C-O-C angle of 112$\vphantom{0}^\circ$^52^, respectively, the monomer length *l* can be calculated using:

3 $$\begin{aligned} l = g+2h = [0.154\, \text {nm} \times \sin (109.5^\circ /2)] + 2[0.143\, \text {nm} \times \sin (112^\circ /2)] = 0.36\, \text {nm}. \end{aligned}$$

As an effective width of the PEO backbone, we consider:

4 $$\begin{aligned} m = 6 \times [0.154\, \text {nm} \times \cos (109.5^\circ /2)]\, = 0.53\, \text {nm}. \end{aligned}$$

This value also agrees with our measurement using the PDB Mol* tool found under https://www.rcsb.org/3d-view. A representative PEG structure model (PubChem CID: 14619693) can be found under^58^. With an ethylene oxide (monomer) molecular weight of 44.05 g/mol and an average molecular weight of 100,000 g/mol for poly(ethylene oxide), the number of monomers amounts to

5 $$\begin{aligned} n = \frac{100,000\,\text {g/mol}}{44.05\, \text {g/mol}} \,= 2270, \end{aligned}$$

which further gives a total length of $L= 2270\, \times 0.36\, \text {nm} = 817\, \text {nm}$. For comparison, PEG4000 (*n*= 91), which is often used as a precipitating agent for the production of protein crystals and/or to stabilize crystals in their aqueous buffer^33,59^, is 33 nm long. Usually, PEG4000 concentrations of 15–20% (w/v) are used for crystal buffers as above 8% (w/v), PEG4000 chains start to overlap and physically entangle, creating elastic properties and increasing the buffer’s viscosity. The entanglement concentration in [g/L] can be calculated with^60^

6 $$\begin{aligned} c_e = 2 \times c^* = 2 \times \frac{M}{8\, N_A\, (R_\text {g}\times 10^{-9})^3} \times 10^{-3} [\text {g/L}], \end{aligned}$$

with the overlap concentration $c^*$, the average molecular weight *M*, and the radius of gyration *R*_g_ in [nm]. To describe the *R*_g_ of polymers in solution, we first consider the chain to be composed of a number $n_\text {K}$ of straight segments each of the length *b* (Kuhn segment length), so the total length can alternatively be described by^61^

7 $$\begin{aligned} L = n_\text {K} \times b \end{aligned}$$

When discussing the degree of entanglement, the polymer’s radius of gyration *R*_g_, which is related to the mean square end-to-end distance $R_0^2$ of a chain,

8 $$\begin{aligned} R_\text {g}^2 = R_0^2 / 6 \end{aligned}$$

with

9 $$\begin{aligned} R_0^2 = Lb = n_K b^2 \end{aligned}$$

is taken into account. The scaling law from Devanand and Selser^62^ allows the calculation of the radius of gyration from empirical data:

10 $$\begin{aligned} R_\text {g} = 0.0215 \times M^{0.583} \, \text {[nm]}, \end{aligned}$$

which provides a *R*_g_ of 17.7 nm for PEO100k and 2.7 nm for PEG4000, respectively. These values are also in agreement with the scaling law from Hofmann *et al.*^63^, that assumes

11 $$\begin{aligned} R_\text {g} = \rho _0 \times L^{\nu }, \end{aligned}$$

with the density $\rho _0$ (*ca.* 1.2 g/mL) and the excluded volume parameter $\nu$, which classifies the chains from being swollen in a good solvent (coil is expanded; $\nu$=0.6) over relaxed (the so-called theta-state; $\nu$=1/2) to partially precipitated in poor solvents (*i.e.* the most compact globule state; $\nu$=1/3), resulting in a *R*_g_ of 34.3 nm for relaxed PEO100k and 6.9 nm for PEG4000, respectively. Therefore, for PEO100k, concentrations above 1% (w/v) lead to entanglement and should be avoided for jet sheathing.

**Fig. 11:**
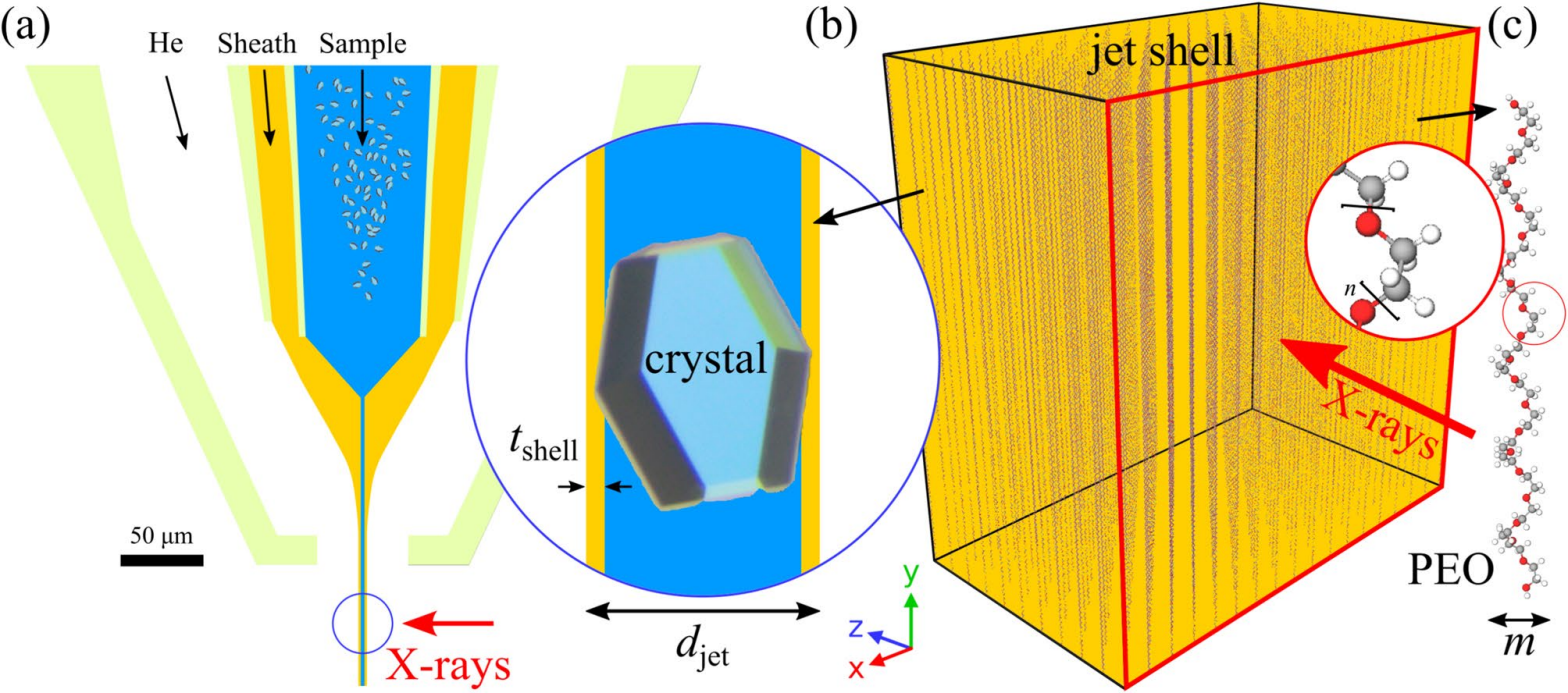
(**a**) Geometry of the ’N9’ DFFN tip with indicated sample channel (aqueous buffer with crystals), sheath liquid channel (PEO solution) and gas inlet. The schematic generation of a compound jet with the diameter *d*_j_, where blue indicates the inner jet diameter $d_\textrm{i} = d_\textrm{j}\sqrt{Q_\textrm{i}/(Q_\textrm{i}+Q_\textrm{o})}$ and orange the shell thickness $t_{\text {shell}} = (d_\textrm{j}-d_\textrm{i})/2$, is indicated. (**b**) Simulated PEO distribution in the jet shell, generated with OVITO^64^. The area of the red rectangle represent the X-ray beam size ($x \times y$) and *z* is the shell’s thickness, which stretches along the beam propagation direction. For clearer visualization, each dimension has been divided by six. (**c**) Structure model of PEG with *n*=21 (PubChem CID: 14619693) as generated with https://molview.org/?cid=14619693^58^. The width *m* of a chain that resides in the jet shell is roughly 0.53 nm (see eq. 4).

As shown in Fig. 11, a single PEO100k chain can roughly fill in a volume of

12 $$\begin{aligned} V_{\text {PEO}} =m^2 \times L = 230\, \text { nm}^3. \end{aligned}$$

Considering a PEO-sheathed jet as depicted in Fig. 4b with $d_\text {j} =$ 3.9 µm and $t_{\text {shell}}$ = 360 nm, the volume, that the X-rays with a beam size of $x_{\text {spot}}$ = 600$\times$600 nm^2^ would hit, is

13 $$\begin{aligned} V_{\text {shell}}=2\times t_\text {shell} \times x_{\text {spot}} = 2.592\times 10^8 \text { nm}^{3}. \end{aligned}$$

The ratio of the shell’s volume to the PEO volume gives the theoretical number of densely packed chains that are present in $V_{\text {shell}}$ at a concentration of 100% (w/v):

14 $$\begin{aligned} V_{\text {shell}}/V_{\text {PEO}} = \frac{2.592\times 10^8 \text { nm}^{3}}{{230 \ \text {nm}^3}} = 1,126,957. \end{aligned}$$

Therefore, our 1% (w/v) solution used for the jetting should provide 11,270 chains that are hit by the X-rays. Figure 11b depicts a simulation of PEO chains in a box of the volume $V_{\text {shell}}/2$, which is one side of the jet shell. The concentration in the x and z directions is: $c_x = c_z = 0.1$. We assume that the PEO molecules are closely packed in the *y* direction: $c_y = 1.0$. The volume concentration amounts to $c = c_x \times c_y \times c_z = 1\%$. In the graphic representation, each dimension has been divided by six for clearer depiction. The total number of chains enclosed in the simulation box in *x* direction is given by

15 $$\begin{aligned} N_x=\frac{600 \ \text {nm}}{0.53 \ \text {nm}}\times 0.1 = 113, \end{aligned}$$

the number of chains enclosed in the simulation box in *z* (beam path) is given by

16 $$\begin{aligned} N_z=\frac{360 \ \text {nm}}{0.53 \ \text {nm}}\times 0.1 = 68, \end{aligned}$$

and the number of chains enclosed in the simulation box in *y* is given by

17 $$\begin{aligned} N_y=\frac{600 \ \text {nm}}{817 \ \text {nm}}\times 1.0 = 0.73, \end{aligned}$$

respectively.

Therefore, there are $N = N_x \times N_y \times N_z = 5647$ chains in the simulation box. When one includes the jet shell on the other side of the core, the total number of chains is 11, 294, which agrees with the estimation below eq. 14.

### PEO stretching and relaxation

The PEO molecules in the shell undergo two successive deformation-relaxation processes that underpin the observed superstability of the jet. (i) As the jet forms at the nozzle, the PEO chains enter the thin meniscus shell in their equilibrium coiled conformation and are rapidly stretched by the strong extensional flow field generated by the accelerating gas flow as it expands past the exit orifice. (ii) After the jet emerges and the external gas shear decreases, the polymer chains begin to relax back toward their coiled state. However, the characteristic relaxation time of the PEO solution is longer than the residence time of fluid elements in a jet governed solely by Newtonian behavior. As a result, the polymer remains partially stretched over the observed jet length, providing a persistent elastic stress that stabilizes the jet against capillary breakup. The observed jet length is therefore consistent with the relaxation dynamics of the stretched PEO molecules. This analysis aligns with^7^, which attributes jet superstability (lengths $>{2}\,\hbox {mm}$, diameters $\sim$2 to 4 µm) to PEO molecule stretching in the tapering meniscus and viscoelastic stabilization in the jet.

#### Gas velocity and jet diameter

Given a gas exit orifice diameter of $D_{\text {gas}} = {60}\,\upmu \hbox {m}$ and a gas mass flow rate of $\dot{m}_g$ = 30 mg/min, and assuming the gas temperature at the nozzle is approximately $T \approx 280\,\text {K}$, the corresponding stagnation pressure for a typical discharge coefficient $C_g=0.7$ in these kind of nozzles is $P_0 = 2.65 \times 10^{5}\,\text {Pa}$, yielding a sonic discharge velocity of about $853\,\text {m/s}$. The associated Bernoulli discharge velocity of the liquid is then $v_l \approx 23\,\text {m/s}$. From Fig. 8, the PEO–water jet diameter is estimated to be ${4.5}\,\upmu \hbox {m}$. For a total liquid flow rate of 45 µL/min the resulting axial jet velocity is approximately $40\,\text {m/s}$, compatible with the additional acceleration due to tangential shear imposed by the co-flowing gas.

#### Shear rate in the jet shell

The shear rate in the jet shell can be calculated from the shear stress at the gas-liquid interface, which arises from the velocity difference between the helium gas (850 m/s at the nozzle exit) and the jet (40 m/s). The initial shear stress on the jet surface is given by:

18 $$\begin{aligned} \tau = \mu _g \left| \frac{du_g}{dy} \right| _{y=0} \approx \mu _g \frac{\Delta v}{\delta _g}, \end{aligned}$$

where $\mu _g = {1.98 \times 10^{-5}}\,\hbox {Pa}\,\hbox {s}$ (helium viscosity at 298 K), $\Delta v = {800}\,{\hbox {m}}\,\hbox {s}^{-1}$, and $\delta _g \approx$ 9.5 µm (estimated average boundary layer thickness). The average thickness of the helium gas boundary layer could be estimated at a traveled distance of 60 µm, equal to the diameter of the orifice, using the Blasius solution for a laminar boundary layer. The density at the choked exit amounts to:

19 $$\begin{aligned} \rho ^* = \frac{P^*}{R T^*} \approx {0.297}\,\hbox {kg}/\hbox {m}^{3}, \end{aligned}$$

with $$P^* = 1.294 \times 10^{5}\,\hbox {Pa}, T^* = {209.9}\,\hbox {K},$$$$\text {and } R = {2077}\,\hbox {J}\,\hbox {kg}^{-1}\,\hbox {K}^{-1}.$$ The dynamic viscosity of helium at 209.9 K is calculated using the Sutherland model:

20 $$\begin{aligned} \mu = \mu _0 \left( \frac{T}{T_0} \right) ^{3/2} \frac{T_0 + S}{T + S} \approx 1.527 \times 10^{-5}\hbox {Pa}\,\hbox {s}, \end{aligned}$$

where $\mu _0 = 1.98 \times 10^{-5}\,\hbox {Pa}\,\hbox {s}$ at $T_0 = {298}\,\hbox {K}$, and $S = {80}\,\hbox {K}$. Thus, the Reynolds number at $x = 6.0 \times 10^{-5}\,\hbox {m}$ is

21 $$\begin{aligned} Re_x \approx 994.6. \end{aligned}$$

Finally, using the Blasius formula for a laminar boundary layer for a travel distance of $x\approx 60$ µm is approximately:

22 $$\begin{aligned} \delta \approx \frac{4.92 x}{\sqrt{Re_x}} \approx {9.5}\,\upmu \hbox {m}. \end{aligned}$$

From previous estimations, the shear stress can be approximated as:

23 $$\begin{aligned} \tau \approx {1.3}\,\hbox {kPa}. \end{aligned}$$

For the thin shell of 360 nm thickness, the shear stress is approximately uniform, equal to the previous estimate. The shear rate, using the shell viscosity ($\eta \approx 1 \times 10^{-3}\,\hbox {Pa}\,\hbox {s}$, adjusted for shear-thinning), is:

24 $$\begin{aligned} \dot{\gamma } \approx \frac{\tau _{\text {shell}}}{\eta } \approx 1.3 \times 10^{6}\,\hbox {s}^{-1}. \end{aligned}$$

Using the Zimm relaxation time of a PEO molecule of approximately $\lambda _r \approx$ 7 ns, this yields a Weissemberg number $Wi =\dot{\gamma } \lambda _r \approx 0.009 \ll 1$, which would indicate that the molecules will relax in the jet once the stretching process in the meniscus is complete. However, this is valid for dilute solutions. At 1 % (w/v) concentration, a relaxation time $\lambda _r=26.2 \,$ µs is considered^7^, which yields $Wi \approx 34$, indicating a strongly delayed relaxation along the jet once the molecules have been stretched in the meniscus.

#### PEO stretching in the meniscus

The extensional strain rate is significantly higher than the shear rate in the meniscus. For uniaxial extensional flow, the extensional strain rate in the tapering meniscus is the axial velocity gradient:

25 $$\begin{aligned} \dot{\epsilon } = \frac{dv}{dz} = -\frac{2Q}{\pi r(z)^3} \frac{dr}{dz}. \end{aligned}$$

Since the strain rate varies along the meniscus, we evaluate it near the tip (where stretching is maximized, as per^7^), at $r(z) \approx r_j = 2.5 \times 10^{-6}\,\hbox {m}$:

26 $$\begin{aligned} r(z)^3 \approx 1.6 \times 10^{-17}\,\hbox {m}^{3}. \end{aligned}$$

Figure 8 suggests a gradient (30 µm in an axial length of 60 µm):

27 $$\begin{aligned} \frac{dr}{dz} \approx -0.5, \end{aligned}$$

which yields, for $Q=7.5 \times 10^{-10}$ m$\vphantom{0}^3$/s,

28 $$\begin{aligned} \dot{\epsilon } \approx {1.5 \times 10^7}\,\hbox {s}^{-1}. \end{aligned}$$

Using a relaxation time ($\lambda _r = {26.2}\,\upmu \,\hbox {s}$) yields a Weissemberg number:

29 $$\begin{aligned} Wi = \dot{\epsilon } \lambda _r \approx 390. \end{aligned}$$

This $Wi \gg 1$ confirms a coil–stretch transition. The time to stretch from the relaxed end-to-end distance ($L_0 \approx \sqrt{3} \times {34.3}\,\hbox {nm} \approx {59.4}\,\hbox {nm}$) to 817 nm is:

30 $$\begin{aligned} t_{\text {stretch}} \approx \frac{\ln (L / L_0)}{\dot{\epsilon }} \approx {200}\,\hbox {ns}, \end{aligned}$$

which occurs within the meniscus residence time ($\sim 2\,\upmu \hbox {s}$).

#### Relaxation in the jet shell

The radius of gyration for PEO ($M_V=$ 100,000 g/mol) in water is $R_g = {17.7}\,\hbox {nm}$. For a Gaussian chain, the coiled end-to-end distance is:

31 $$\begin{aligned} L_0 = \sqrt{6} R_g \approx {43.3}\,\hbox {nm}. \end{aligned}$$

In the jet shell, the shear rate is $\dot{\gamma } \approx 1.3 \times 10^{6}\,\hbox {s}^{-1}$ (with boundary layer thickness of 9.36 µm), yielding:

32 $$\begin{aligned} Wi = \dot{\gamma } \lambda _r \approx 34. \end{aligned}$$

From the FENE(Finitely Extensible Nonlinear Elastic)-dumbell model, the steady-state extension under shear is:

33 $$\begin{aligned} L_{\text {steady}} \approx L_0 \sqrt{2 Wi} \approx {358}\,\hbox {nm}. \end{aligned}$$

This $L_{\text {steady}}$ is significantly closer to the extended (or total) length ($L_{\text {total}} = {817}\,\hbox {nm}$) than to the coiled state ($L_0 = {43.3}\,\hbox {nm}$). The extension ratio relative to the coiled state is approximately 8.3, while relative to the extended length is approximately 0.44, indicating that the polymer is extended to only 44% of its maximum length, far beyond its equilibrium coil size. The elastic energy stored in the polymer is proportional to the square of the extension, as per the FENE model:

34 $$\begin{aligned} E_{\text {elastic}} \propto \frac{R^2}{L_{\text {contour}}^2 - R^2}, \end{aligned}$$

where $R \approx L_{\text {steady}}$. For $L_{\text {steady}} = {358}\,\hbox {nm}$, this energy is significant due to the large extension relative to $L_0$, reflecting a high degree of chain alignment induced by the shear flow. This stored energy enhances the viscoelastic properties of the 360 nm-thick shell, increasing extensional viscosity and stabilizing the jet against perturbations, as evidenced by the extended jet length compared to Newtonian jets^7^. Interestingly, the shell thickness is here approximately equal to the steady stretched length. This suggests that stretched PEO chains, aligned along the direction of maximum principal stress, are confined within the thin annular shell, potentially forming a load-bearing molecular network that enhances jet superstability. In fact, the principal stress, nearly axial, orients molecules along the $z$-axis with a slight tilt due to $\sigma _{rz}$. The angle $\phi$ of molecular alignment relative to the axis can be estimated from the shear-induced velocity gradient across the shell as less than ${1}^\circ$, indicating that PEO chains are nearly parallel to the jet axis, with minimal helical deviation, confined within the 360 nm shell thickness. The partial extension is further sustained by hypothesized entanglement or self-assembly of PEO chains (Figs. 7,8 in^7^), which delays relaxation beyond the intrinsic $\lambda _r$. Even in the absence of this effect, from the Zimm model for polymer relaxation, the time to relaxation near the coiled state ($L(t) \approx 1.05 \times L_0 \approx {45}\,\hbox {nm}$) can be written as:

35 $$\begin{aligned} L(t) = L_{\text {steady}} + (L_{\text {total}} - L_{\text {steady}}) e^{-t / \lambda _r}, \end{aligned}$$

with $L_{\text {total}}=817$ nm, which yields

36 $$\begin{aligned} t \approx {10.1}\,\upmu \hbox {s}. \end{aligned}$$

With these models, the jet length traveled (at $v_j = {40}\,\hbox {m}\,\hbox {s}^{-1}$) is: ${40}\,\hbox {m}\,\hbox {s}^{-1} \times 10.1 \times 10^{-6}\,\hbox {s} \approx {0.4}\,\hbox {mm}.$ This relaxation length is shorter than the observed jet length, suggesting that the polymer remains partially extended throughout much of the jet and stores elastic energy as mentioned. Given that the gas speed decreases significantly beyond some orifice diameters downstream, the $L_{\text {steady}}$ further decrease along the jet axis, releasing the elastic energy stored. The released elastic energy in the PEO shell opposes Rayleigh-Plateau instability by enhancing extensional viscosity and damping radial perturbations. In effect, elastic tension in the shell, anchored at core-shell and shell-gas interfaces, counteracts radial pinching, delaying breakup. The drag effect, potentially amplified by entanglement or self-assembly^7^, increases the effective viscosity, suppressing instability growth. In conclusion,

- Stretching in the meniscus occurs in $\sim$226 ns ($Wi \approx 340$), consistent with the coil-stretch transition in^7^.
- Relaxation in the jet shell to near the coiled state (45.5 nm) takes $\sim$10 µ s ($\sim$0.5 mm), shorter than the observed jet length ($\sim$2 mm, 42 µ s residence time).
- Jet stability is enhanced by residual elastic stress and possible entanglement/self-assembly, as hypothesized in^7^ (Figs. 7,8), bridging the gap between 0.4 mm and about 2 mm. This elastic energy storage is a key mechanism underlying the superstability of micrometer-scale jets, enabling applications in serial femtosecond X-ray crystallography.

## Materials and methods

### Sample preparation

#### Poly(ethylene oxide)

**Fig. 12:**
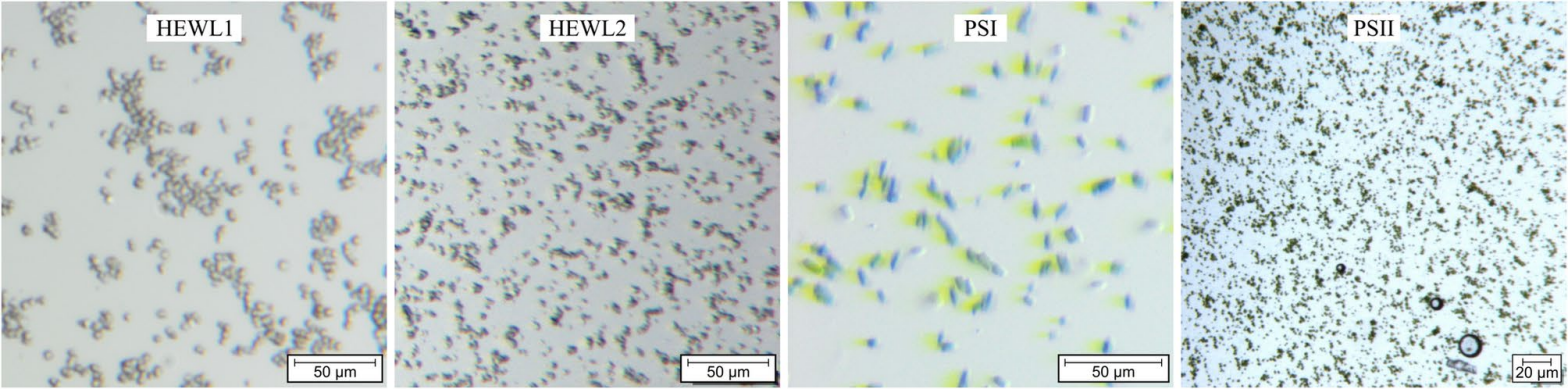
Optical microscopy images of 2–4 µm large HEWL, 5–10 µm PSI and *ca.* 4 µm PSII microcrystals in their aqueous buffers. The buffer for HEWL1 is composed of 10% (w/v) NaCl and 50 mM sodium acetate (pH 3.5). HEWL2 additionally contains 20% (w/v) PEG 6000. PSI contained 5 mM MES (pH 6.4) and 0.02% (w/v) *n*-dodecyl-*ß*-D-maltoside. PSII contained 50 mM PIPES (pH 7.0), 5 mM CaCl_2_, 7% (w/v) PEG1450 and 25% (w/v) PEG6000.

PEO100k (Sigma-Aldrich, 181986), with an average molecular weight of 100,000 g/mol (*M*_v_), was dissolved in dH_2_O to provide a 1% (w/v) solution. The solution was filtered through a Luer syringe compatible nylon cell sieve with 10 µm pore sizes (neoCulture, C-8239) (Fig. 12).

#### Hen egg white lysozyme (HEWL)

’HEWL1’ samples (experiment no. 8796) were prepared according to^2^. The crystals with 2–4 µm in diameter were dispensed in 10% (w/v) NaCl and 50 mM sodium acetate (pH 3.5) and further filtered through a 20 µm nylon mesh (CellTrics). The crystal concentration after sedimentation (pellet) was *ca.* 20% (v/v) as judged by the volume comparison from pellet to supernatant. A concentration of 7$\times$10^8^ crystals/mL was roughly estimated using a Neubauer chamber (improved). During injection, an inline filter with 30$\times$34 µm^2^ mesh openings was utilized. For a more viscous version of this sample (’HEWL2’), the same crystals were dispersed in 10% (w/v) NaCl, 50 mM sodium acetate (pH 3.5) and 20% (w/v) PEG 6000.

#### Photosystem I

PSI (experiment no. 3111) was crystallized by the rapid dilution with seeding method as described in^65^. For the sample no. 11 (S4-P4) that was used in experiment no. 3111, 8 µL of a photosystem I solution containing 80 mg/mL protein (20 mM chlorophyll concentration) in 5 mM MES pH (6.4), 50 mM MgSO_4_ and 0.02% (w/v) *n*-dodecyl-*ß*-D-maltoside (DDM) were rapidly diluted with a low ionic strength buffer (5 mM MES pH 6.4, 0.025% (w/v) DDM) that contained 0.5 nm large PSI seeds. The chlorophyll concentration of the seeded dilution buffer was 20 µM. The final chlorophyll concentration of the crystallization solution was 0.3 mM. After running the crystal slurry through a nylon gravity filter (30 µm wide meshes, CellTrics, 04–0042−2316), crystal sizes of 5–10 µm were determined.

#### Photosystem II

PSII samples for experiment no. 8756 were prepared according to^17^and crystallized using the microbatch method as previously reported^19^. An aliquot of 5 µm PSII crystals (unfiltered) was delivered using an inline filter with 50$\times$71 µm^2^ mesh openings. The crystal concentration after settling in the delivery buffer was estimated to be 10% (v/v) and the buffer was composed of 50 mM PIPES (pH 7.0), 5 mM CaCl_2_, 7% (w/v) PEG1450 and 25% (w/v) PEG6000.

### SFX

The SFX data (proposal no. 8796) were collected on the scientific instrument SPB/SFX^66^ of the European XFEL (Schenefeld, Germany) using X-rays with a photon energy of 11 keV ($\lambda$ = 0.113 nm) arriving in 10 Hz trains with 352 pulses per train and an intra-train pulse repetition rate of 1.13 MHz. As a detector, the 1 Mpixel adaptive gain integrating pixel detector (AGIPD 1M) with a pixel size of 200 µm $\times$200 µm was used^67^. With a sample-to-AGIPD distance of 124.3 mm, *q* ranged from 3.5 to 39.8 nm^−1^ at detector half-width (resolution: 18.1-1.6 Å), where *q* = 4$\pi$/$\lambda$ sin($\theta$) is the length of the scattering vector and $\theta$ is the half-scattering angle. The beam diameter was adjusted to 600 nm in the horizontal (*x*) direction and 600 nm in the vertical (*y*) direction (FWHM) using nano-KB mirrors^68^. The de-focusing of the mirrors led to a beam size of 3.5 µm $\times$ 3.5 µm (Fig. 2).

For illuminating the jets during SFX, a visible light laser (Litron, Nano SG 150-10) with a wavelength of 532 nm (output energy = 50 mJ), a pulse length of 5–7 ns, and a pulse repetition rate of 10 Hz was used. The spot size at the nozzle was ca. 10 mm. The side view microscope was a Zyla 5.5 sCMOS equipped with a 10$\times$ objective (Mitutoyo MY10X-803, 0.28 NA) leading to a pixel size of 0.65 µm and a theoretical Abbe’s optical resolution of 0.95 µm. An experimental resolution of 1.38 µm was revealed by imaging a USAF resolution target. The side-microscopy images were captured at a 10 Hz collection rate. The optical laser was timed in such a way as to arrive right after the second X-ray pulse, hence showing two gaps in the liquid column.

SFX data processing was performed using the CrystFEL package (Version 0.11.1)^69^ via EXtra-Xwiz data processing pipeline^70^. Bragg peaks were detected using peakfinder8^24^ and frames were indexed using XGANDALF^71^. The entire peak search and index processing was performed identically across all data sets. A resolution cut-off of 1.7 Å (4.5 Å for PSII) for all sets was applied to display statistics with at least >95% completeness in the outer shells and a minimum redundancy of 20 measurements per unique reflection (except for PSII). The crystallography results are shown in Tables 3 and 4. Solution scattering patterns were averaged using geomtools^72^ and azimuthally integrated using pyFAI^73^ within the EXtra-geom library^74^.

### XRD

Laboratory-based X-ray diffraction was performed on a Bruker D8 Advance device (8.04 keV) equipped with a LYNXEYE XE-T detector with 191 channels. The powders and aqueous solutions were analyzed at 23 $\vphantom{0}^\circ$C in rotating 1.5 mm wide borosilicate glass (3.3) capillaries (Hilgenberg). 2$\theta$ scans from 10 to 50$\vphantom{0}^\circ$ were collected with a step width of 0.01$\vphantom{0}^\circ$ and an exposure time of 10 s, multiplied with the 191 channels. The scattering curves were analyzed with GSAS-II^75^ and revealed signals at 2$\theta$=19.12$\vphantom{0}^\circ$ (120), 21.11$\vphantom{0}^\circ$ (031), 21.57$\vphantom{0}^\circ$ (023), 22.04$\vphantom{0}^\circ$ (121), 23.00$\vphantom{0}^\circ$ (112/004), and 23.25$\vphantom{0}^\circ$ (032) as well as the unit cell parameters *a* = 8.03 (literature^52^: 8.05) Å, *b* = 13.09 (13.04) Å, *c* = 19.41 (19.48) Å, and $\beta$ = 125.1$\vphantom{0}^\circ$ (125.4$\vphantom{0}^\circ$) for crystalline PEO100k (monoclinic-P lattice with space group *P*2_1_/*a*).

### Rheology

Dynamic viscosities were determined with an MCR92 rheometer (Anton Paar) in combination with a CP35-0.5 (SN82632) probe for shear rates between 0.5 and 10,000 1/s.

## Data Availability

Data recorded for the experiment 8796 at the European XFEL are available at 10.22003/XFEL.EU-DATA-008796-00 upon reasonable request.
